# Stepwise Evaluation of Nasal Breathing After Functional Rhinofiller: A Prospective Peak Nasal Inspiratory Flow-Based Study

**DOI:** 10.1093/asjof/ojag168

**Published:** 2026-09-12

**Authors:** Matteo Basso, Emanuela Di Lella, Manuele Casale, Simone Di Giovanni, Giovanni Cristalli, Francesco Marchetti

## Abstract

**Background:**

Nonsurgical rhinoplasty with hyaluronic acid (HA) fillers is widely used for aesthetic nasal correction; however, its potential impact on nasal respiratory function remains underexplored. Structural alterations of the nasal tip and columella may influence the external nasal valve and nasal airflow dynamics.

**Objectives:**

To prospectively evaluate the functional effects of a two-step HA rhinofiller technique on nasal breathing using objective and subjective assessment tools, and to determine the individual contribution of columellar and nasal tip augmentation.

**Methods:**

Nine adult female patients with functional nasal breathing impairment related to reduced tip support underwent a standardized two-step rhinofiller procedure. Nine adult female patients seeking nonsurgical rhinofiller treatment for aesthetic nasal concerns, with concomitant varying degrees of subjective nasal obstruction, underwent a standardized two-step rhinofiller procedure. Sequential HA injections were performed at the columella and nasal tip. Objective nasal airflow was measured using peak nasal inspiratory flow (PNIF) at baseline, after the first injection, and after the second injection. Subjective breathing quality was assessed using a visual analog scale (VAS) at baseline, at 10 days, and at 60 days.

**Results:**

Mean PNIF increased significantly after columellar injection (+17.7%) and showed a further significant improvement following nasal tip augmentation (+5.4%), resulting in an overall PNIF increase of 24.0% compared with baseline. Subjective VAS scores improved significantly at 10 days and remained significantly higher than baseline at 60 days. No complications were observed.

**Conclusions:**

A targeted two-step functional rhinofiller protocol using HA can significantly improve nasal airflow and patient-perceived breathing quality. Columellar augmentation appears to provide the greatest functional benefit, supporting the concept of rhinofiller as a minimally invasive functional intervention in selected patients.

**Level of Evidence: 2 (Therapeutic):**

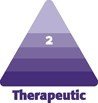

The nose plays a central role in both facial aesthetics and respiratory function, representing the primary conduit for nasal airflow. Alterations in nasal tip projection and rotation, whether congenital or age-related, may contribute not only to an unaesthetic appearance but also to functional impairment by modifying the geometry of the nasal vestibule and external nasal valve. In particular, reduced tip support and caudal rotation can increase nasal resistance, negatively affecting inspiratory airflow.^1-3^

Surgical rhinoplasty remains the gold standard for addressing both aesthetic and functional nasal deformities; however, nonsurgical rhinoplasty with hyaluronic acid (HA) fillers has gained increasing popularity as a minimally invasive alternative. HA rhinofiller allows controlled modulation of nasal shape, including improvement of tip projection, rotation, and columellar support, with minimal downtime and the advantage of reversibility through hyaluronidase.^1,2,4-6^ While the aesthetic outcomes of rhinofiller are well documented, its potential functional impact on nasal breathing has only recently gained attention.

From a physiological perspective, nasal airflow is highly dependent on the configuration of the nasal valve area, the columella–tip complex, and the spatial relationship between the lower lateral cartilages. Structural reinforcement at the level of the columella and nasal tip may improve airflow by stabilizing the external nasal valve and optimizing vestibular patency. Conversely, inappropriate filler placement or excessive volume could theoretically worsen nasal resistance, highlighting the importance of precise technique and objective functional assessment.^5,6^

Objective evaluation of nasal breathing can be performed using rhinomanometry or peak nasal inspiratory flow (PNIF). PNIF is a simple, reproducible, and cost-effective bedside test that correlates well with subjective nasal obstruction and other objective airflow measurements PNIF is a simple, inexpensive, and reproducible measure of global nasal airflow that has been widely validated in patients with nasal obstruction. Previous studies have demonstrated significant correlations between PNIF values, patient-reported nasal obstruction, rhinomanometry, and clinical outcomes following functional nasal interventions. Although less commonly used in aesthetic surgery practice, PNIF offers a practical bedside assessment tool that allows objective evaluation of changes in nasal airflow without the need for specialized laboratory equipment.^7^ Previous studies have demonstrated that rhinofiller may improve nasal airflow; however, available data are largely retrospective and do not clarify the individual contribution of specific injection sites to functional improvement.^5,8^

Therefore, the aim of the present study was to prospectively evaluate the functional impact of a two-step functional rhinofiller technique using HA, specifically designed to improve nasal breathing. By performing sequential injections at the level of the columella and subsequently at the nasal tip, and by measuring PNIF before treatment, after the first injection, and after the second injection, we sought to assess the individual and cumulative effects of each anatomical intervention on nasal airflow. Secondary outcomes included subjective assessment of breathing quality using a visual analog scale (VAS). Through this stepwise approach, the present study aims to clarify the role of targeted columellar and tip support in improving nasal respiratory function.^1-8^

## METHODS

### Study Design and Patient Selection

This prospective observational study included adult patients seeking nonsurgical rhinofiller treatment primarily for functional improvement of nasal breathing. This prospective observational study included adult female patients seeking nonsurgical rhinofiller treatment primarily for aesthetic nasal correction. All enrolled patients presented with varying degrees of subjective nasal obstruction, generally mild in severity, although the primary reason for consultation was aesthetic rather than functional. The study was designed to investigate whether structural support of the nasal tip and columella through rhinofiller treatment could also influence nasal airflow. Procedures were performed in a single private clinic by the same experienced injector, in accordance with standard aseptic techniques. Inclusion criteria were age ≥18 years and the presence of functional nasal breathing impairment associated with reduced nasal tip support or rotation. Patients with previous nasal surgery, significant septal deviation, turbinate hypertrophy, chronic rhinosinusitis, active nasal infection, known hypersensitivity to hyaluronic acid, or clinically significant internal nasal valve insufficiency requiring surgical correction were excluded. In general, patients considered candidates for surgical treatment were not included in the study. All participants provided written informed consent prior to enrollment. According to local regulations, Institutional Review Board approval was not required for this observational study involving noninvasive aesthetic procedures. The study was conducted in accordance with the Declaration of Helsinki.

### Injection Technique

A standardized two-step functional rhinofiller technique using hyaluronic acid (HA) was performed with the specific aim of improving nasal breathing through targeted structural and dynamic support.

The filler used in all patients was Neauvia® Intense (Matex Lab, Switzerland), a cross-linked hyaluronic acid with a concentration of 28 mg/mL, characterized by high elasticity (G′), high cohesivity, and suitability for deep structural support. These rheological properties make it particularly appropriate for nasal applications requiring mechanical stability and resistance to dynamic forces.

The first injection was administered at the level of the columella, in correspondence with the anterior nasal spine region rather than the mid-columella. Approximately 0.05 mL of HA was injected in the deep midline plane between the medial crura. This injection was designed to provide structural support to the columella–tip complex and to reduce the downward traction exerted by the depressor septi nasi muscle. By reinforcing this area, the procedure aimed to limit excessive tip descent during inspiration and facial animation, thereby contributing to improved nasal airflow stability.

The second injection was subsequently performed at the level of the nasal tip, in the supratip/interdomal region between the tip-defining points and in relation to the medial crura and lower lateral cartilages. Approximately 0.05 mL of HA was injected in the deep subcutaneous or supracartilaginous plane using a slow microbolus technique. This step was intended to enhance tip projection and rotation, resulting in improved opening of the external nasal valve and optimization of air entry during inspiration.

The total injected volume did not exceed approximately 0.10 mL per patient. All injections were performed using a needle, with careful aspiration and slow delivery to minimize vascular risk. No cannulas were used All injections were performed using a 30-gauge needle to allow precise placement of small filler aliquots in the intended deep midline anatomical planes; needle use was not considered a specific measure to reduce vascular risk. Careful aspiration was performed prior to injection, and the product was delivered in a controlled manner while maintaining a stable depth throughout the procedure. Because only very small volumes of a highly cohesive hyaluronic acid filler were used (approximately 0.05 mL per injection site), the total injected volume remained limited. The authors acknowledge that vascular compromise remains a potential complication of all nasal filler procedures, regardless of whether a needle or cannula is used, and that meticulous anatomical knowledge, careful patient selection, and appropriate informed consent are essential. Video 1 includes embedded captions indicating the relevant anatomical landmarks and injection planes (Figure 1; Video 1).

**Figure 1.**
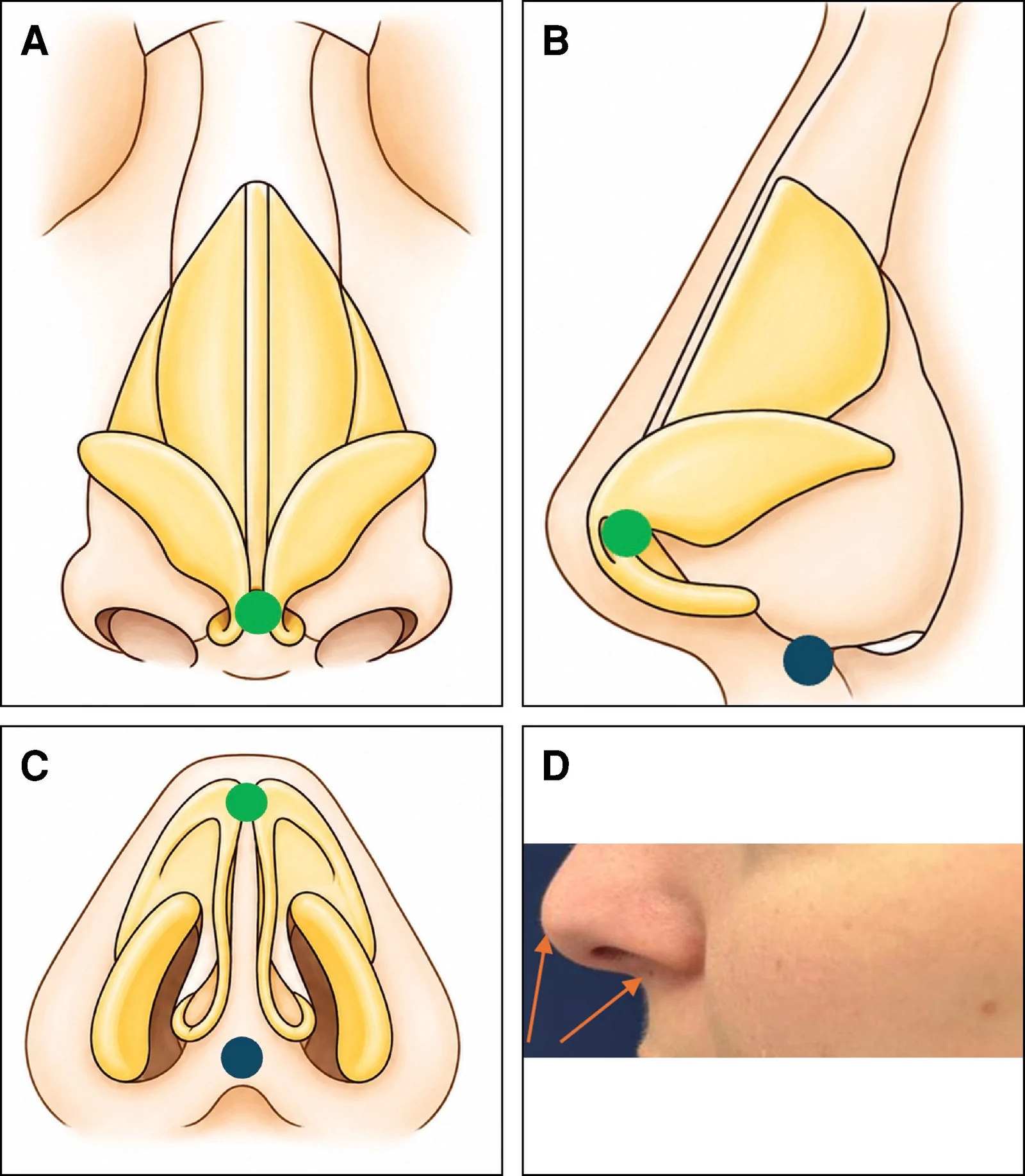
Injection sites for the two-step functional rhinofiller technique. (A–C) Schematic anatomical illustrations of the injection sites in (A) frontal, (B) lateral, and (C) basal views. The marker positioned at the supratip/interdomal region indicates the nasal tip injection site, whereas the marker positioned at the columellar base near the anterior nasal spine indicates the second injection site. These injection sites are intended to enhance tip projection, improve columellar support, and optimize nasal valve function. (D) Representative lateral clinical view of a 23-year-old female patient, with arrows indicating the corresponding treatment areas.

Objective nasal airflow was assessed using a PNIF meter (Clement Clarke International, UK) (Figure 2). Measurements were performed with the patient in an upright sitting position, at three time points: before treatment (T0), immediately after the first columellar injection (T1), and immediately after the second nasal tip injection (T2). At each time point, patients performed three maximal inspiratory efforts, and the highest value, expressed in liters per minute (L/min), was recorded for analysis (Video 2). Subjective nasal breathing was assessed using a VAS at baseline, at 10 days, and at 60 days following the procedure to evaluate both short-term and early mid-term patient-reported outcomes.

**Figure 2.**
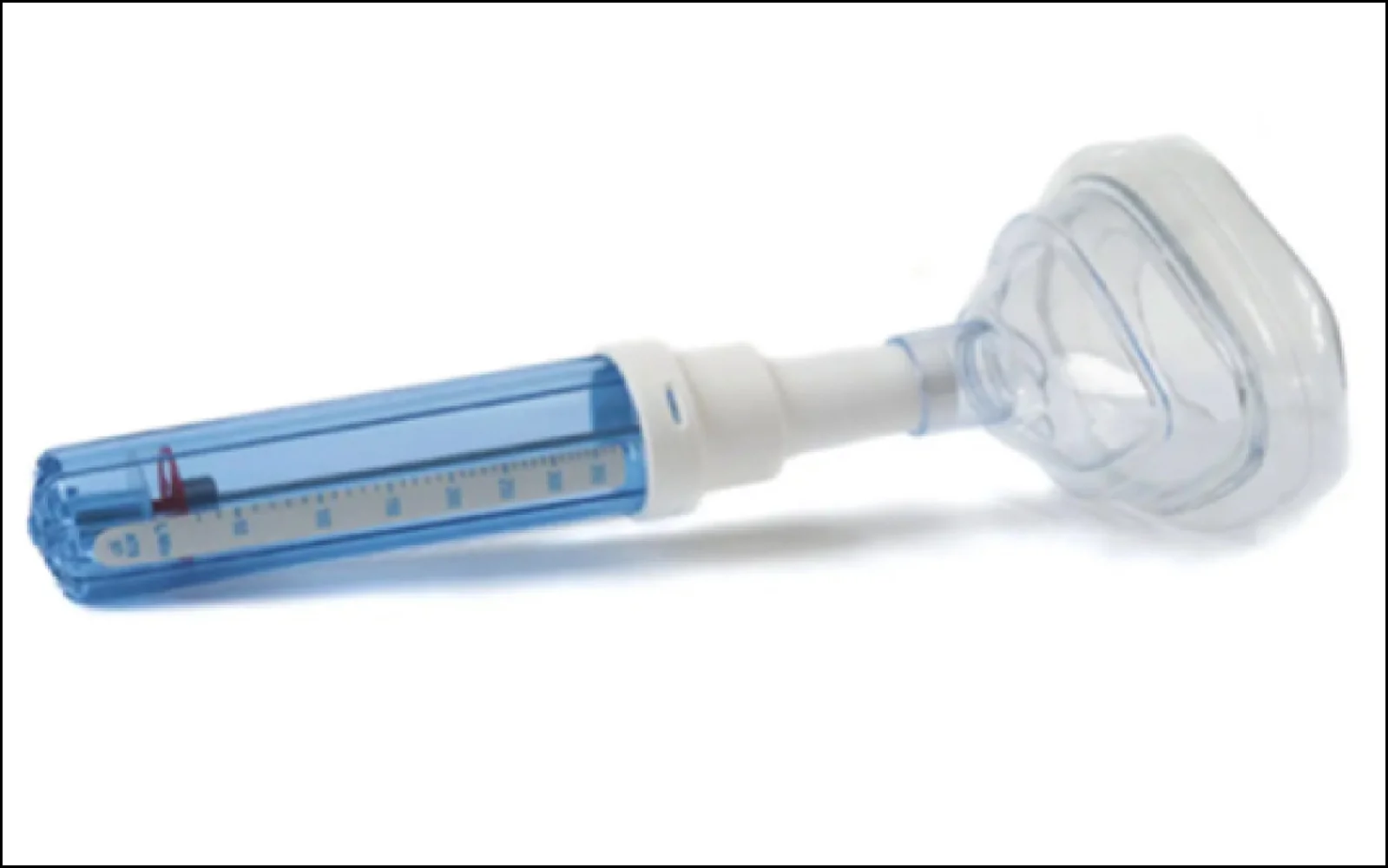
Peak nasal inspiratory flow (PNIF) meter. Handheld PNIF device (Clement Clarke International, UK) used for objective nasal airflow assessment in the present study.

### Statistical Analysis

Statistical analysis was performed using dedicated statistical software (GraphPad Prism, GraphPad Software, San Diego, CA). Changes in objective and subjective nasal breathing parameters were evaluated over time. PNIF values, recorded at baseline, after the first columellar injection, and after the second nasal tip injection, were analyzed using repeated-measures statistical methods. Data distribution was assessed for normality, and comparisons among time points were performed using appropriate parametric or nonparametric tests for paired data.

Subjective nasal breathing assessed by the VAS was compared between baseline and follow-up using paired statistical analysis. Continuous variables are presented as mean ± SD. All tests were two-tailed, and a *P*-value <.05 was considered statistically significant.

## RESULTS

The study included 9 female patients with a mean age of 38.6 ± 13.8 years (range 23-64 years). All patients completed the study protocol, and no complications were observed during the study period.

At baseline, objective nasal airflow assessed by PNIF showed a mean value of 87.8 ± 25.4 L/min, while subjective nasal breathing evaluated using the VAS demonstrated a mean score of 7.33.

Following the first injection at the level of the columella, mean PNIF increased to 103.3 ± 25.0 L/min, corresponding to an absolute increase of **+**15.5 L/min and a relative improvement of **+**17.7% compared with baseline. This increase was statistically significant (Wilcoxon signed-rank test, *P* = .0039).

After the second injection at the level of the nasal tip, mean PNIF further increased to 108.9 ± 24.7 L/min. This corresponded to an additional absolute increase of **+**5.6 L/min and a relative improvement of **+**5.4% compared with post–first injection values (Wilcoxon signed-rank test, *P* = .0152). Overall, the combined effect of the two-step rhinofiller procedure resulted in a total PNIF increase of **+**21.1 L/min, corresponding to a **+**24.0% improvement compared with baseline (Table 1). Representative lateral photographs illustrating improved nasal tip projection and rotation following the two-step procedure are presented in Figure 3.

**Figure 3.**
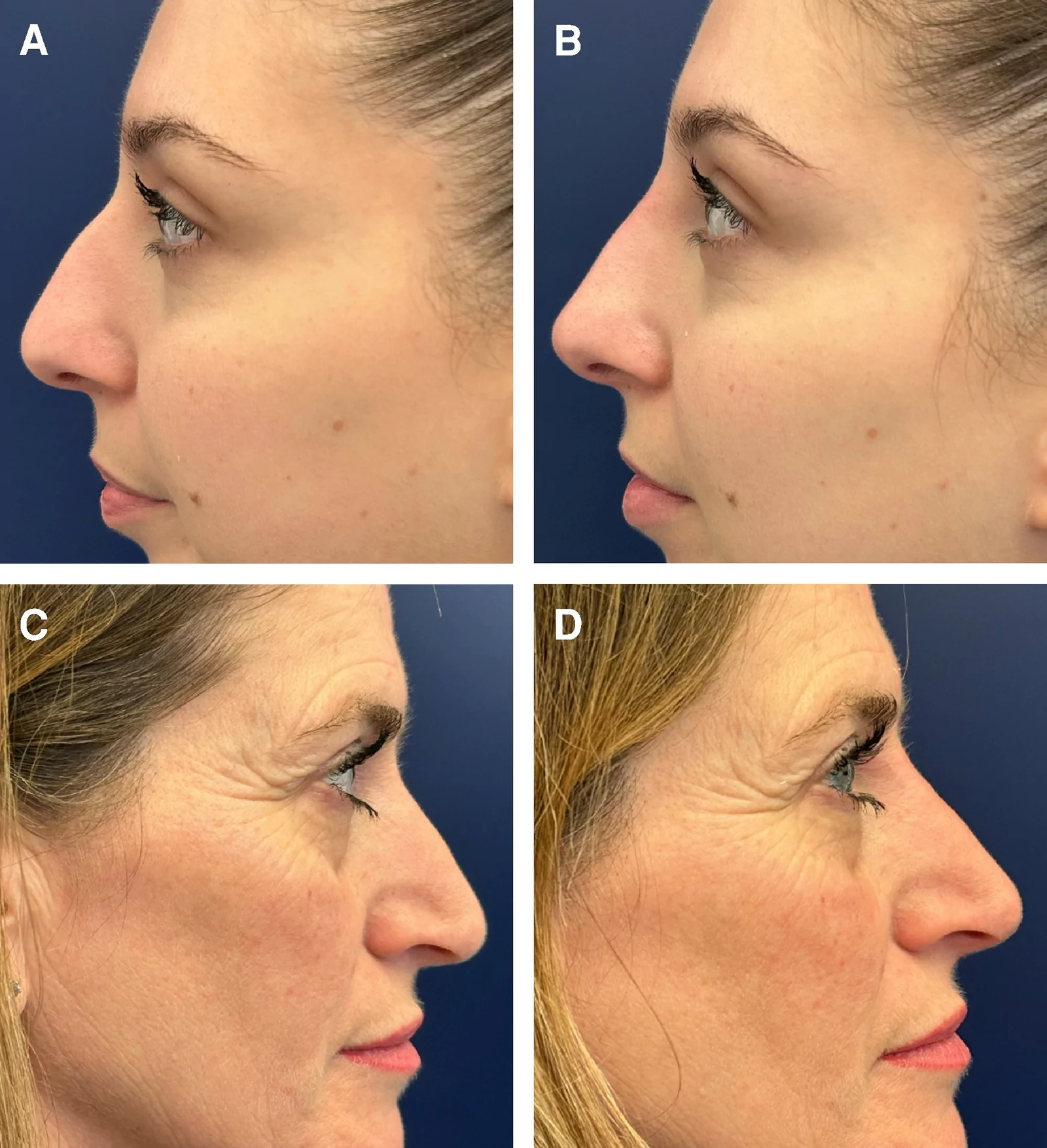
Representative lateral views before and after the two-step functional hyaluronic acid rhinofiller procedure. Improvement in nasal tip projection and rotation is shown following sequential columellar and nasal tip augmentation with hyaluronic acid. (A, B) A 23-year-old female patient (A) at baseline and (B) after treatment. (C, D) A 51-year-old female patient (C) at baseline and (D) after treatment.

**Table 1.** Objective and Subjective Nasal Breathing Outcomes Following a Two-Step Functional Rhinofiller Procedure

Parameter	Comparison/time point	Mean ± SD	Absolute change	Percentage change	*P*-value
PNIF (L/min)	Baseline (T0)	87.8 ± 25.4	—	—	—
	Post 1st injection (T1)	103.3 ± 25.0	+15.5 (vs T0)	+17.7% (vs T0)	.0039
	Post 2nd injection (T2)	108.9 ± 24.7	+21.1 (vs T0)	+24.0% (vs T0)	—
	Increment T1 → T2	—	+5.6 (vs T1)	+5.4% (vs T1)	.0152
VAS (breathing)	Baseline (T0)	7.33 ± 0.71	—	—	—
	10 days follow-up	8.44 ± 0.52	+1.11 (vs T0)	+15.1% (vs T0)	.0083
	60 days follow-up	8.33 ± 0.87	+1.00 (vs T0)	+13.6% (vs T0)	<.01
	10 → 60 days	—	−0.11 (vs 10 days)	−1.3% (vs 10 days)	.32

Peak nasal inspiratory flow (PNIF, L/min) was measured at baseline (T0), after columellar injection (T1), and after nasal tip injection (T2). Subjective nasal breathing was assessed using a visual analog scale (VAS) at baseline, at 10 days, and at 60 days follow-up to evaluate short-term and early mid-term patient-reported outcomes. Data are presented as mean ± SD. Statistical analysis was performed using the Wilcoxon signed-rank test.

Subjective nasal breathing, assessed using VAS, showed a significant improvement after treatment. The mean VAS score increased from 7.33 ± 0.71 at baseline to 8.44 ± 0.52 at 10 days (*P* = .0083), and remained significantly higher than baseline at 60 days (8.33 ± 0.87, *P* < .01). A slight reduction was observed between 10 and 60 days; however, this difference was not statistically significant (*P* = .32) (Table 1).

## DISCUSSION

A growing body of literature has demonstrated that nonsurgical rhinoplasty using hyaluronic acid (HA) fillers may influence not only nasal aesthetics but also nasal respiratory function. Several clinical studies, technical reports, and systematic reviews have described improvements in nasal airflow, patient-reported breathing comfort, or both following rhinofiller procedures targeting the dorsum, columella, and nasal tip. Objective functional assessments using rhinomanometry, acoustic rhinometry, and PNIF have further supported the concept that structural reinforcement of key nasal regions can positively affect nasal aerodynamics.^7-10^ Collectively, these data suggest that rhinofiller should not be regarded exclusively as an aesthetic procedure, but rather as a potential functional intervention in carefully selected patients.

The present prospective study confirms this general trend, demonstrating a statistically significant improvement in nasal breathing following a functional rhinofiller protocol, documented by both objective (PNIF) and subjective (VAS) measures.

A growing body of literature has demonstrated that nonsurgical rhinoplasty using hyaluronic acid (HA) fillers may influence not only nasal aesthetics but also nasal respiratory function. Several clinical studies, technical reports, and systematic reviews have described improvements in nasal airflow, patient-reported breathing comfort, or both following rhinofiller procedures targeting the dorsum, columella, and nasal tip. Objective functional assessments using rhinomanometry, acoustic rhinometry, and PNIF have further supported the concept that structural reinforcement of key nasal regions can positively affect nasal aerodynamics.^7-10^

In the present study, PNIF was selected as the primary objective outcome measure because it represents a simple, inexpensive, and readily applicable method for assessing nasal airflow in routine clinical practice. Previous studies have demonstrated correlations between PNIF values, patient-reported nasal obstruction, and rhinomanometric findings, supporting its use as a practical objective measure of nasal patency.^7,9,10^ At the same time, we acknowledge the well-recognized limitations of PNIF reported in the literature. As a global assessment of inspiratory nasal airflow, PNIF does not identify the anatomical site of obstruction and may be influenced by patient effort, motivation, and other individual factors. For this reason, objective PNIF measurements were complemented by subjective VAS assessments, providing a combined evaluation of both airflow changes and patient-perceived breathing improvement. Importantly, this study introduces a novel methodological aspect by evaluating nasal airflow sequentially after each injection. To the best of our knowledge, previous studies assessing the functional impact of rhinofiller have relied on pre- and post-treatment comparisons, without isolating the contribution of individual injection sites on respiratory outcomes.^8-11^

One of the most relevant findings of this study is that the first injection, performed at the level of the columella, produced the greatest functional improvement, while the second injection at the nasal tip resulted in an additional but smaller increase in airflow. In our cohort, PNIF increased by 17.7% after columellar augmentation and by a further 5.4% after nasal tip injection. This observation suggests that reinforcement of the columella–tip complex may exert a greater influence on nasal airflow than tip projection alone. From a functional standpoint, deep midline columellar augmentation increases rigidity of the medial crura and caudal septal support, reducing dynamic tip descent and improving the stability of the external nasal valve during inspiration. In contrast, nasal tip injection mainly enhances projection and rotation, contributing to external nasal valve opening but with a more limited incremental effect once baseline structural support has been restored.^1,4,5,12^

These findings are consistent with established principles of nasal airflow physiology, whereby small changes in the geometry and stability of the nasal valve region can lead to significant variations in airflow resistance.^7,13^ In surgical functional rhinoplasty, maneuvers aimed at reinforcing the caudal septum, columella, and nasal valve are considered critical determinants of respiratory outcome. The present results suggest that targeted HA augmentation may partially reproduce similar biomechanical effects in a minimally invasive and reversible manner, without altering osseocartilaginous structures.^5,12,14^

When compared with the available literature, this study adds a relevant contribution by providing a stepwise functional evaluation of rhinofiller. Calvani et al^8^ demonstrated that rhinofiller can improve nasal airflow using active anterior rhinomanometry and subjective questionnaires, introducing the concept of “functional rhinofiller”. However, their retrospective design did not allow assessment of the individual contribution of specific injection sites. Similarly, technique-oriented studies focusing on nasal tip reshaping and Italian rhinofiller approaches have demonstrated reliable aesthetic and morphometric outcomes but did not include objective respiratory measurements.^1,4^ Systematic reviews on nonsurgical rhinoplasty confirm the increasing use and safety of HA nasal injections, while underlining the limited availability of standardized functional and site-specific data.^2,5,6^

From a clinical perspective, the stepwise approach adopted in this study may have important implications. The results suggest that prioritizing columellar support may be particularly effective when the primary goal is functional improvement, potentially reducing the need for additional injections and minimizing overall filler volume. Moreover, sequential PNIF assessment could represent a simple periprocedural tool to guide treatment decisions and to identify patients who may benefit from further nasal tip support. PNIF has been widely validated as a reliable, reproducible, and clinically practical method for assessing nasal airflow and correlates well with subjective perception of nasal obstruction.^7,10^

Importantly, subjective VAS scores remained significantly improved at 60 days compared with baseline, with no statistically significant decline between 10 and 60 days. While these data do not replace objective long-term airflow measurements, they provide preliminary evidence that the perceived functional benefit may extend beyond the immediate postprocedural period. This finding is encouraging, although confirmation through serial objective assessments over longer follow-up intervals remains necessary.

These findings align with recent evidence supporting the persistence of functional rhinofiller outcomes. Calvani et al^8^ reported sustained rhinomanometric improvement at 6 months, while Barone et al^15^, in a larger cohort of 121 patients, confirmed functional gains on NOSE scale and rhinomanometry up to 6 months. Gagnieur et al^16^ further demonstrated persistent nasal valve improvement at 1 year following endonasal HA injection. Together with the present VAS data at 60 days, these results suggest that the functional benefit of targeted HA augmentation may extend beyond the immediate postprocedural period, although long-term objective confirmation using PNIF remains necessary.

From an anatomical and safety perspective, the nasal tip and columella represent a complex vascular territory supplied by branches of the superior labial artery and lateral nasal artery.^17,18^ Filler injection in this region carries inherent risks, including vascular compromise, skin necrosis, and, in rare cases, ophthalmic complications. In the present study, no adverse events were observed; however, the small sample size limits the ability to draw definitive safety conclusions. We therefore avoid interpreting needle use, aspiration, or slow product delivery as definitive methods for preventing vascular compromise. Rather, the choice of a 30-gauge needle was based on the need for accurate placement of very small aliquots in the intended deep midline anatomical planes. A thorough understanding of the local vascular anatomy and the spatial relationship between the lower lateral cartilages, the columellar artery, and the nasal tip vasculature is essential for safe practice. Future studies should include a structured safety assessment with systematic documentation of complications such as bruising, swelling, asymmetry, and vascular events, to provide a comprehensive safety profile of the functional rhinofiller technique. This exploratory study was designed to assess stepwise functional changes following a targeted rhinofiller technique rather than to provide definitive population-level estimates. The primary aim was to evaluate the relative functional contribution of individual injection steps using objective and subjective measures. This study has several limitations. The sample size was small and consisted exclusively of female patients, and ethnicity was not recorded or analyzed; given that nasal anatomy, skin thickness, and cartilage structure vary significantly across ethnic groups, this homogeneity may limit the generalizability of the findings. The objective functional assessment (PNIF) was limited to the immediate periprocedural period, and longer-term PNIF measurements were not obtained. Although subjective VAS data at 60 days suggest persistence of the perceived functional benefit, objective confirmation of airflow improvement beyond the day of treatment is lacking. Serial objective measurements at 1 week, 1 month, 3 months, and 6 months would be required to characterize the temporal evolution of functional outcomes and to establish the durability of the observed benefits. Standardized serial photographic documentation, including basal views, was not performed; this limits the morphological assessment of the procedure and the evaluation of potential columellar widening over time. In addition, although PNIF is a practical and widely used objective measure, it reflects global inspiratory airflow and may be influenced by patient effort and transient procedural factors. Finally, the small sample size limits the ability to draw definitive conclusions regarding the safety profile of the technique. Future prospective studies involving larger and more diverse populations, extended follow-up with objective measurements, standardized photographic protocols, and the incorporation of complementary anthropometric assessments are warranted to better delineate the functional impact of specific rhinofiller injection sites and to refine patient selection criteria.

### Limitations

This study has several limitations. The sample size was small and consisted exclusively of female patients, potentially limiting the generalizability of the findings. Moreover, the study population primarily consisted of patients seeking aesthetic nasal correction, although all participants also reported varying degrees of subjective nasal obstruction. Therefore, the applicability of these findings to patients primarily seeking treatment for functional nasal obstruction remains uncertain.

The objective functional assessment (PNIF) was limited to the immediate periprocedural period, and longer-term PNIF measurements were not obtained. Although subjective VAS data at 60 days suggest persistence of the perceived functional benefit, objective confirmation of airflow improvement beyond the day of treatment is lacking. Because 60 days was the longest follow-up in the present study, the duration of the functional effects cannot be established. Given the resorbable nature of hyaluronic acid fillers, these effects should not be considered long-term at this stage and may prove to be transient, potentially requiring periodic retreatment to maintain the observed benefits. Serial objective measurements at 1 week, 1 month, 3 months, and 6 months would be required to characterize the temporal evolution of functional outcomes and establish the durability of the observed benefits. In addition, the absence of a control group limits the ability to determine whether the observed improvements were exclusively attributable to the intervention. Standardized serial photographic documentation, including basal views, was not performed; this limits the morphological assessment of the procedure and the evaluation of potential columellar widening over time. Furthermore, although PNIF is a practical and widely used objective measure, it reflects global inspiratory airflow and may be influenced by patient effort and transient procedural factors. Finally, the small sample size limits the ability to draw definitive conclusions regarding the safety profile of the technique.

Future prospective studies involving larger and more diverse populations, extended follow-up with objective measurements, standardized photographic protocols, and the incorporation of complementary anthropometric assessments are warranted to better delineate the functional impact of specific rhinofiller injection sites and refine patient selection criteria.

## CONCLUSIONS

A two-step functional rhinofiller protocol using hyaluronic acid resulted in a significant improvement in nasal breathing, as demonstrated by objective PNIF measurements and subjective VAS scores. Columellar augmentation provided the greatest functional benefit, with nasal tip injection yielding an additional but smaller improvement. These findings support the role of targeted rhinofiller as a minimally invasive option for selected patients with reduced nasal tip support and concomitant subjective nasal breathing impairment. However, this approach should not be regarded as a substitute for surgical septorhinoplasty when structural nasal obstruction requires operative correction.
